# Supplementary figures and images for: Environmental and anthropic factors influencing Aedes aegypti and Aedes albopictus (Diptera: Culicidae), with emphasis on natural infection and dissemination: Implications for an emerging vector in Colombia
Source: PLoS Negl Trop Dis. 2025 Apr 8;19(4):e0012605. doi: 10.1371/journal.pntd.0012605 (PMC12077778; doi:10.1371/journal.pntd.0012605)

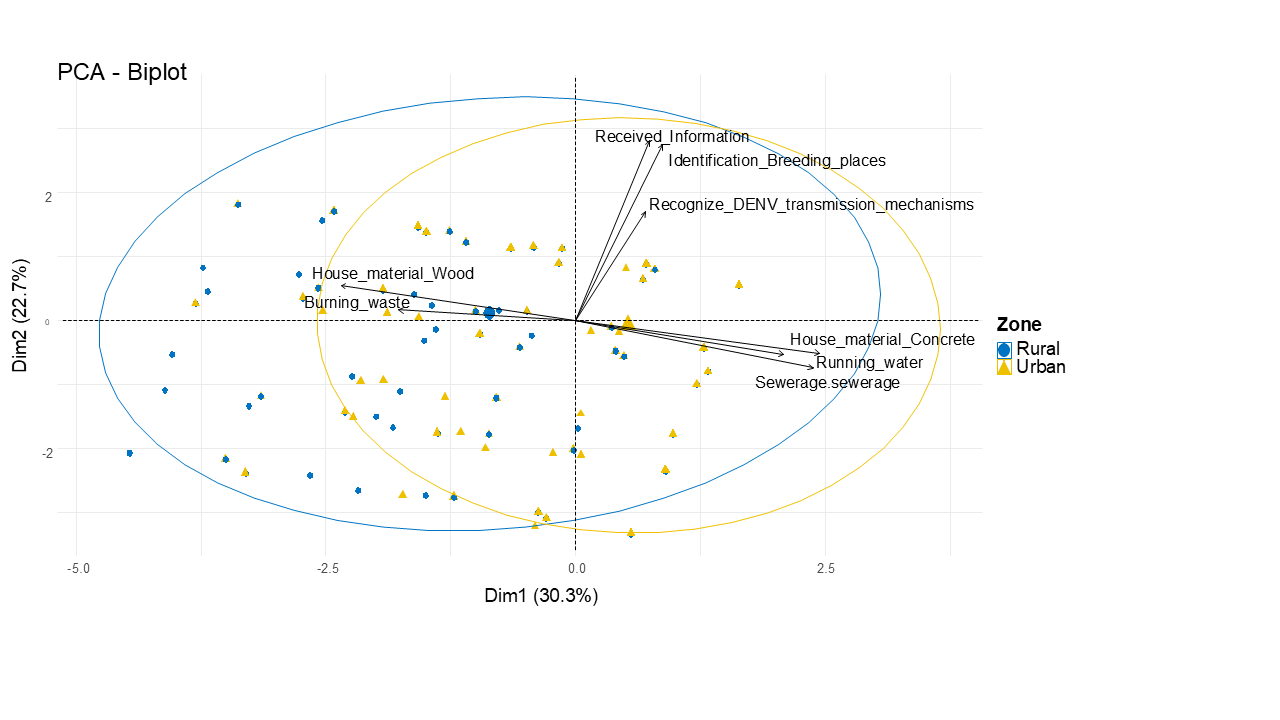

Supplement: S1 Fig — The best-represented variables at each component were selected according to the eigenvalues. The points represent sampled houses at urban (yellow triangles) and rural (blue points). (TIFF) [file pntd.0012605.s001.tif]
