# Supplementary material for: Environmental and anthropic factors influencing Aedes aegypti and Aedes albopictus (Diptera: Culicidae), with emphasis on natural infection and dissemination: Implications for an emerging vector in Colombia
Source: PLoS Negl Trop Dis. 2025 Apr 8;19(4):e0012605. doi: 10.1371/journal.pntd.0012605 (PMC12077778; doi:10.1371/journal.pntd.0012605)
Supplement: S1 Table — (DOCX) [file pntd.0012605.s002.docx]

S1. Summarized data of urban and rural areas per sampled municipality.

|  | **Factor** | Piamonte | | Patía | | Piendamó | | Popayán | |
| --- | --- | --- | --- | --- | --- | --- | --- | --- | --- |
|  |  | **Urban** | **Rural** | **Urban** | **Rural** | **Urban** | **Rural** | **Urban** | **Rural** |
| Climatic | Altitude (masl) | 232-346 | 301-477 | 605-1024 | 609-1168 | 1328-1906 | 1679-1942 | 1817-1932 | 1703-2165 |
|  | Maximum temperature (°C) | 30 (28-30.8) | 30.3(29.3-31) | 30.3 (28.8-31) | 30.3 (28-33.3) | 23.8 (22.5-24.8) | 25.1 (22.5-26) | 23 (22-25) | 22.6 (19.5-25) |
|  | Minimum temperature (°C) | 20.1 (19-21) | 20.1 (20-21) | 18.3 (18-18.5) | 18.4(17.5-20) | 12.2 (12-13) | 13.5 (12.5-14) | 11.8(11.5-13) | 11.5 (8.8-13) |
|  | Precipitation (mm) | 461 (315-563) | 452(306-567) | 190.8 (131-272) | 196 (135-277) | 190 (120-311) | 225 (158-327) | 177 (64-306) | 174 (64-306) |
| Sampled | Sampled houses | 122 | 43 | 156 | 83 | 142 | 62 | 146 | 150 |
|  | Resampled houses | 83 | 33 | 113 | 62 | 81 | 42 | 115 | 113 |
|  | Inhabitant per house* | 3.6(1-8) | 3.5(1-7) | 3.6(1-9) | 3(1-9) | 3.6(1-9) | 3.8(1-9) | 3.5(1-8) | 4.1(1-11) |
| House conditions | Water containers per house (number of containers) * | 1.3(0-4) | 1.3 (0-4) | 1.2(0-5) | 1.3(1-4) | 1.3(1-5) | 1.6(1-5) | 1.1(1-3) | 1.6(0-7) |
|  | House material (concrete) (Number of houses) | 50 | 4 | 153 | 72 | 128 | 58 | 127 | 106 |
|  | House material (wood) (Number of houses) | 72 | 39 | 3 | 8 | 10 | 3 | 12 | 33 |
|  | House material (other) (Number of houses) | 0 | 0 | 0 | 6 | 7 | 2 | 7 | 11 |
|  | Running water (Number of houses) | 89 | 21 | 129 | 74 | 103 | 0 | 136 | 34 |
|  | Irregular running water service (Number of houses) | 96 | 31 | 130 | 33 | 4 | 34 | 0 | 16 |
|  | Water storage (Number of houses) | 81 | 23 | 114 | 71 | 35 | 50 | 25 | 66 |
|  | Rainwater collection (Number of houses) | 2 | 1 | 0 | 0 | 0 | 0 | 0 | 4 |
|  | Trash disposal service (Number of houses) | 122 | 41 | 154 | 82 | 142 | 61 | 145 | 143 |
|  | Burying waste (Number of houses) | 0 | 1 | 0 | 1 | 0 | 19 | 0 | 3 |
|  | Burning waste (Number of houses) | 0 | 18 | 3 | 6 | 11 | 37 | 2 | 57 |
|  | Electricity (Number of houses) | 120 | 40 | 132 | 83 | 115 | 60 | 142 | 149 |
|  | Sewerage (Number of houses) | 43 | 0 | 116 | 57 | 93 | 0 | 135 | 12 |
| Knowledge | Reporting received previous information about dengue | 88 | 23 | 117 | 60 | 100 | 40 | 80 | 100 |
|  | Identification of *Ae. aegypti* potential breeding places | 100 | 31 | 134 | 69 | 119 | 46 | 111 | 116 |
|  | Recognize dengue transmission mechanisms | 108 | 30 | 132 | 67 | 113 | 45 | 117 | 123 |
|  | Recognized who can get infected by dengue | 109 | 33 | 140 | 65 | 106 | 40 | 119 | 114 |
|  | Do not know *Ae. aegypti* potential breeding places | 13 | 5 | 13 | 4 | 18 | 4 | 4 | 7 |
| *Aedes aegypti* | Number of larvae** | 132 (0.64) | 1 (0.01) | 511 (1.9) | 259 (1.8) | 22(0.09 | 0 | 34 (0.1) | 34(0.1) |
|  | Number of pupae** | 49 (0.2) | 0 | 300.1 | 29(0.2) | 0 | 0 | 10 (0.04) | 1 (0.003) |
|  | Number of females** | 43 (0.2) | 0 | 131 (0.48) | 60 (0.41) | 13 (0,05) | 0 | 22 (0.08) | 1 (0.003) |
| *Aedes albopictus* | Number of larvae** | 18(0.08) | 99 (1.3) | 78 (0.3) | 27 (0.2) | 4 (0.01) | 21 (0.2) | 2 (0.007) | 29(0.1) |
|  | Number of pupae** | 0 | 14 (0.2) | 9 (0.03) | 0 | 2 (0.009) | 2 (0.02) | 0 | 9 (0.03) |
|  | Number of females** | 9 (0.04) | 0 | 12 (0.04) | 22 (0.15) | 0 | 0 | 8 (0.03) | 3 (0.01) |

* Average and range are given per sampled house ** Total collected and average (per house)
