# Supplementary material for: Environmental and anthropic factors influencing Aedes aegypti and Aedes albopictus (Diptera: Culicidae), with emphasis on natural infection and dissemination: Implications for an emerging vector in Colombia
Source: PLoS Negl Trop Dis. 2025 Apr 8;19(4):e0012605. doi: 10.1371/journal.pntd.0012605 (PMC12077778; doi:10.1371/journal.pntd.0012605)
Supplement: S2 Table — (DOCX) [file pntd.0012605.s003.docx]

Table S2. Summarized information of water containers inspected in urban and rural areas, emphasizing positive breeding places for *Ae. aegypti* and *Ae. albopictus.*

| Area | Type of Container | Number of positive containers (%) | | Total inspected |
| --- | --- | --- | --- | --- |
|  |  | *A. aegypti* | *A. albopictus* |  |
| Rural | Wash basin | 23 (4.8) | 9 (1.9) | 481 |
|  | Buckets | 6 (3) | 2 (1) | 200 |
|  | Drain sumps | 0 | 0 | 1 |
|  | Tires | 5 (10) | 9 (18) | 50 |
|  | Others | 3 (3.3) | 4 (4.4) | 90 |
|  | Ground tanks | 5 (6.8) | 1 (1.4) | 74 |
|  | Bamboos | 0 | 0 | 2 |
| Urban | Wash basin | 59 (7.1) | 7 (0.8) | 833 |
|  | Buckets | 21 (11.4) | 7 (3.8) | 184 |
|  | Drain sumps | 1 (33.3) | 2 (66.7) | 3 |
|  | Tires | 4 (20) | 4 (20) | 20 |
|  | Others | 10 (11.2) | 3 (3.4) | 89 |
|  | Ground tanks | 8 (8.5) | 1 (1.1) | 94 |
|  | Bamboos | 0 | 1 (50) | 2 |
