# Supplementary material for: Environmental and anthropic factors influencing Aedes aegypti and Aedes albopictus (Diptera: Culicidae), with emphasis on natural infection and dissemination: Implications for an emerging vector in Colombia
Source: PLoS Negl Trop Dis. 2025 Apr 8;19(4):e0012605. doi: 10.1371/journal.pntd.0012605 (PMC12077778; doi:10.1371/journal.pntd.0012605)
Supplement: Attachment — Submitted filename: Answering letter D-24-01455 manuscript.docx [file pntd.0012605.s005.docx]

Editor-in-Chief
PLOS Neglected Tropical Diseases

Subject: Response to Reviewers’ Comments for Manuscript [PNTD-D-24-01455]

Dear Editor and reviewers,

Thank you for the opportunity to revise our manuscript [PNTD-D-24-01455]. We greatly appreciate the constructive feedback provided by the reviewers, which has helped us improve our work. Below, we provide a detailed point-by-point response to each reviewer's comments, highlighting the revisions made. Changes in the manuscript have been marked for clarity.

**Reviewer #1:**

The objectives are well achieved. The design study was appropriate to achieve objectives. The vector population is correctly described and accomplished. The sample and the statistical analysis were adequate to reach the conclusions. There are not any concerns about ethical and regulatory requirements.

Answer: We appreciate the positive feedback regarding our study's objectives, design, statistical analysis, and ethical considerations.

**Reviewer #2:**

I do think that objectives and methods are clearly articulated. However, some improvements of the writing should be done in order to fully understand them. Some of my main concerns are the following:

1.- I understand that localities were chosen by considering their elevation and consequently their temperature range differs. However, how is their endemicity status? I think authors should include information about this in the description of the study area as it can bias the infection results.

Answer

Endemicity status of study sites:
We have included information on the endemicity status of each locality in the study area description (lines 126-128):
"According to Colombia's national classification for dengue transmission (2021–2023), Piamonte, Piendamó, and Popayán are categorized as low transmission risk areas, while Patía is classified as having a medium transmission risk."

2.- How were sampled households selected? Was randomizing software used?

Answer

*Household selection method:*
We have clarified the household selection process in the Methods section (lines 138-144):
"This cross-sectional study used the household as the unit of analysis, with the sample size determined using projected population data from the national census (<https://www.dane.gov.co/>) and calculated using the SSPropor tool (<http://www.openepi.com/>). Households were selected based on their altitude and previous reports of dengue cases or mosquito presence, as provided by the health authorities of each municipality."

3.- I suggest improving the writing so the categorization into artificial and natural containers in clearer

Answer

Container categorization:
We have revised the distinction between artificial and natural containers for clarity (lines 152-160):
"Containers were categorized as either artificial or natural. Artificial containers, defined as man-made objects capable of holding water, included laundry sinks, ground tanks (storage tanks with a capacity >2000 L placed on the ground), tires, buckets, and drain sewers. Less common artificial containers were classified under the category 'others.' Natural containers, defined as naturally occurring structures or formations that collect water, included bamboo, aquatic plants, tree holes, and plant axils. This classification was applied to containers found in three spatial areas: intradomicile (inside the household), peridomicile (0–10 m from the household), and extradomicile (>10 m from the household) [27]."

4.- Please specify what Zeiss ™ Stemi ™ DV4 is. Is it a stereo microscope?

Answer

*Zeiss Stemi™ DV4:* We have specified that this equipment is a stereo microscope. (Line 170)

5.- What period of time was used to collect climatic data from WorldClim? It’s important to mention if the climatic data corresponds well with the time of collection. Otherwise, why was such data used?

*Climatic data time period:* is now specify as follows: line 193-194: “The average, minimum, and maximum temperature values, along with total precipitation for the collection periods”.

6.- Explain further how the principal components analysis was done? The vast majority of the section “Anthropogenic variables” are results. I would strongly suggest to rewrite this section and leave only methods here and move the remaining to Results.

Answer

*Principal Component Analysis (PCA):* The methodology for PCA is now explained in greater detail Line 199-223. Additionally, we have moved results from the “Anthropogenic Variables” Line 259-278 section into the Results section to ensure consistency in structure.

7.- It is not clear to me how authors estimate the risk of mosquito biting from density of females data. I strongly suggest to explain this beyond the inclusion of a reference.

Answer

*Mosquito biting risk estimation:* We have expanded our explanation in to the Entomological index section Lines 185-190:” For adult mosquitoes, we calculated the Female infestation rate – Number of female mosquitoes collected per household during a 15-minute Prokopack aspirator sampling in intradomicile areas. Additionally, the females-per-person index was calculated by dividing the female infestation rate per household by the number of household inhabitants.  Mosquito biting risk was evaluated as the potential contact between mosquitoes and humans, based on the results of pupae-per-person and female-per-person indexes”.

8.- Are in these areas circulating mosquito species other than the two mentioned in the manuscript? E.g. culex species? If so, how authors clean the data related to residents’ responses about their knowledge on Aedes from those of other species?

Answer

*Other mosquito species in the area:* We acknowledge the presence of other mosquito species, such as Culex spp. We now explain how we ensured that resident responses specifically pertained to Aedes species as follows in lines 206-209: “ To prevent confusion with other mosquito species, local health authorities were consulted regarding commonly used names for the adult and immature stages of dengue vectors. Residents were also asked about the "white-striped mosquito" to distinguish it from *Culex* species.”

**Reviewer #3:**

*1.- Clarification on Aedes albopictus as a vector in Colombia:*

Answer

We now emphasize why the vector status of A. albopictus in Colombia is under question despite its known global role. Additional justification has been added in the Introduction in lines 100-105: “However, aspects of vector competence, such as the capacity for arbovirus dissemination in field-collected *Ae. albopictus* populations from endemic countries like Colombia, remain largely understudied. This knowledge gap is particularly critical, as because environmental factors, local socio-economic conditions, and mosquito genetic variability could influence its ability to transmit arboviruses such as DENV, CHIKV, and ZIKV.”

2.- *Table 2:*

We have moved Table 2 to the supplementary material.

*3.- Public knowledge data:*

We acknowledge that this section may seem out of focus. However, we justify its inclusion in the Discussion by explaining its relevance to vector control strategies.

*4.- Disseminated infection in Aedes albopictus:*

The distinction in processing methods between A. aegypti and A. albopictus is now explicitly stated: Lines 173-177: “Since the dissemination capacity of Colombian *Ae. albopictus* populations has not been evaluated, females were dissected into the midgut, legs, and thorax, and each part was preserved individually in RNAlater. Males and immature mosquitoes of both species were processed similarly to *Ae. Aegypti”*.

Table 4 has been renamed to reflect that it specifically refers to A. albopictus Line 419 “**Table 3. Arbovirus molecular detection in legs/Torax or salivary glands from  *Aedes albopictus*  field collected females”.**

**Results Section:**

1. *Household revisits:* We now indicate how many houses were visited twice in table S1.
2. *Biting risk estimation:* As noted earlier, we have clarified the methodology behind this estimation to ensure its biological relevance lines 185-190.
3. *DENV and CHIKV pool positivity:* It seems strange that is similar but is not a typing errors.
4. *ZIKV detection in males:* We have re-examined and confirmed these results, ensuring that cross-reaction or contamination was not an issue, additionally we stated in methodology section line 244:  “nuclease free water as negative control of reaction”.

Additionally, RNA from mosquitoes samples were extracted at different time and areas than positive controls.

**Conclusions Section:**

1. *Discussion improvements:* We have revised the Discussion to enhance clarity, ensuring that broader patterns are more easily identifiable.

**Editorial and Data Presentation Modifications:**

1. *Abbreviations for Culicidae taxonomy:* We have ensured that taxonomic abbreviations align with Reinert’s 2009 guidelines. As *Ae. aegypti* and *Ae. albopictus.*
2. *Figure 1:* We have included additional maps where each locality is shown separately for better visualization.
3. *Minor editorial revisions:* We have addressed minor issues such as replacing “cero” with “zero” (line 196), rewording lines 205-206 for clarity, and defining “high” and “low” house conditions. The term “R0” has also been correctly defined as the basic reproduction number.
4. *Speculative comments on Mayaro virus:* These have been removed as suggested.
5. *Grammatical and typographical corrections:* We have thoroughly reviewed and improved the manuscript’s language.

**Summary and General Comments:**

1. *Geographical characteristics of Cauca:* We now provide additional context on the topography and urban-rural gradient to clarify the distinction between study zones and their potential connectivity, given more detailed maps in figure 1.
2. *Conciseness and broader relevance:* We have streamlined certain sections to make the manuscript more accessible to a broader audience while maintaining key findings.
3. *Manuscript flow and clarity:* We have restructured certain sections to ensure the study’s purpose and key findings remain clear. Statements that overreach conclusions have been adjusted in lines 253-230: “Overall, our results demonstrate that integrating environmental, virological, and anthropogenic variables is crucial for the eco-epidemiological characterization of Aedes-borne viruses, especially in areas where two vector species with distinct ecological characteristics coexist. This approach contributes to a broader understanding of the geographic and temporal dynamics of these arboviruses.”.

**Journal Requirements:**

1) We do not publish any copyright or trademark symbols that usually accompany proprietary names, eg ©, ®, or TM (e.g. next to drug or reagent names). Therefore please remove all instances of trademark/copyright symbols throughout the text, including:
- ® on page: 11 line 220 and line 222.
- TM on page: 8 line 151 and line 153.

response:

we remove the trademark and copyright symbols.

2) Please upload all main figures as separate Figure files in .tif or .eps format. For more information about how to convert and format your figure files please see our guidelines:

<https://journals.plos.org/plosntds/s/figures>

response:

We now upload all the main figures as tif files.

3) The file inventory includes files for Figures 3a, and 3b. We would recommend either combining these into a single Figure 3.tiff file with separate internal panels, or renumbering them as individual figures, and as we are not able to publish multiple components of a single figure as separate files.

response: we rename the figures 3a and 3b ads 3 and 4 figures.

4) Tables should not be uploaded as individual files. Please remove these files and include the Tables in your manuscript file as editable, cell-based objects. For more information about how to format tables, see our guidelines:

<https://journals.plos.org/plosntds/s/tables>

response: we remove tables separated files.

5) We have noticed that you have uploaded Supporting Information files, but you have not included a complete list of legends. Please add a full list of legends including (Paper data file )for your Supporting Information files after the references list.

response: we now add a full list of legends after reference list, for supporting information.

7) Some material included in your submission may be copyrighted. According to PLOSu2019s copyright policy, authors who use figures or other material (e.g., graphics, clipart, maps) from another author or copyright holder must demonstrate or obtain permission to publish this material under the Creative Commons Attribution 4.0 International (CC BY 4.0) License used by PLOS journals. Please closely review the details of PLOSu2019s copyright requirements here: PLOS Licenses and Copyright. If you need to request permissions from a copyright holder, you may use PLOS's Copyright Content Permission form.
Please respond directly to this email and provide any known details concerning your material's license terms and permissions required for reuse, even if you have not yet obtained copyright permissions or are unsure of your material's copyright compatibility. Once you have responded and addressed all other outstanding technical requirements, you may resubmit your manuscript within Editorial Manager.
Potential Copyright Issues:
i) Figure 1. Please (a) provide a direct link to the base layer of the map (i.e., the country or region border shape) and ensure this is also included in the figure legend; and (b) provide a link to the terms of use / license information for the base layer image or shapefile. We cannot publish proprietary or copyrighted maps (e.g. Google Maps, Mapquest) and the terms of use for your map base layer must be compatible with our CC BY 4.0 license.
Note: if you created the map in a software program like R or ArcGIS, please locate and indicate the source of the basemap shapefile onto which data has been plotted.
If your map was obtained from a copyrighted source please amend the figure so that the base map used is from an openly available source. Alternatively, please provide explicit written permission from the copyright holder granting you the right to publish the material under our CC BY 4.0 license.
If you are unsure whether you can use a map or not, please do reach out and we will be able to help you. The following websites are good examples of where you can source open access or public domain maps:
* U.S. Geological Survey (USGS) - All maps are in the public domain. ([http://www.usgs.gov](http://www.usgs.gov/))
* PlaniGlobe - All maps are published under a Creative Commons license so please cite u201cPlaniGlobe, [http://www.planiglobe.com](http://www.planiglobe.com/), CC BY 2.0u201d in the image credit after the caption. (<http://www.planiglobe.com/?lang=enl>)
* Natural Earth - All maps are public domain. (<http://www.naturalearthdata.com/about/terms-of-use/>).

**response**:

we now state in the figure 1 legend: Base layer maps for country departments, urban and rural areas were obtained from open files of: <https://geoportal.dane.gov.co/>, and contour line for elevations were calculated in ArcGis Pro, from digital elevation models downloaded at: [http://www.usgs.gov](http://www.usgs.gov/)_._

8) We note that your Data Availability Statement is currently as follows: "The data supporting the findings of this study have been uploaded as a .txt file and are publicly available." Please confirm at this time whether or not your submission contains all raw data required to replicate the results of your study. Authors must share the “minimal data set” for their submission. PLOS defines the minimal data set to consist of the data required to replicate all study findings reported in the article, as well as related metadata and methods (<https://journals.plos.org/plosone/s/data-availability#loc-minimal-data-set-definition>).
For example, authors should submit the following data:
1) The values behind the means, standard deviations and other measures reported;
2) The values used to build graphs;
3) The points extracted from images for analysis..
Authors do not need to submit their entire data set if only a portion of the data was used in the reported study.
If your submission does not contain these data, please either upload them as Supporting Information files or deposit them to a stable, public repository and provide us with the relevant URLs, DOIs, or accession numbers. For a list of recommended repositories, please see <https://journals.plos.org/plosone/s/recommended-repositories>.
If there are ethical or legal restrictions on sharing a de-identified data set, please explain them in detail (e.g., data contain potentially sensitive information, data are owned by a third-party organization, etc.) and who has imposed them (e.g., an ethics committee). Please also provide contact information for a data access committee, ethics committee, or other institutional body to which data requests may be sent. If data are owned by a third party, please indicate how others may request data access.

**Response:** yes we upload as txt file, the data used for the analysis.

9) Please amend your detailed Financial Disclosure statement. This is published with the article. It must therefore be completed in full sentences and contain the exact wording you wish to be published.
1) State the initials, alongside each funding source, of each author to receive each grant. For example: "This work was supported by the National Institutes of Health (####### to AM; ###### to CJ) and the National Science Foundation (###### to AM)."

**Response**

we changed as follows: This project was funded by Minciencias and Universidad El Bosque contracts 891-2019, 489-2021 to JSM, DSS, MLV, CAM, and JEC, and call 909 de 2021 to JSM and DSS. The funding sources had no role in study design, data collection, analysis, or preparation of the manuscript.

We thank the reviewers for their valuable feedback and believe that these revisions have substantially improved our manuscript. We appreciate your consideration and look forward to your response.

Sincerely,
